# A Cross-Sectional Study Exploring Postpartum Depression at a Tertiary Care Center in Eastern Uttar Pradesh, India

**DOI:** 10.7759/cureus.58653

**Published:** 2024-04-20

**Authors:** Babita Kapoor, Najma Malik, Geeta Gupta, Imran Ahmed Khan

**Affiliations:** 1 Obstetrics and Gynecology, Maharshi Devraha Baba Autonomous State Medical College, Deoria, IND; 2 Obstetrics and Gynecology, Baba Raghav Das Medical College, Gorakhpur, IND; 3 Obstetrics and Gynecology, Autonomous State Medical College, Kushinagar, IND; 4 Community Medicine, Baba Raghav Das Medical College, Gorakhpur, IND

**Keywords:** risk factors, pregnancy, postpartum, edinburgh postnatal depression scale, depression

## Abstract

Background

Postpartum depression (PPD) is a significant public health concern globally characterized by a spectrum of mood disturbances ranging from mild mood swings to severe depressive episodes initiating within four weeks post childbirth and potentially persisting up to 12 months. Besides affecting the mother, it also affects the mental health and development of the babies born to affected mothers. Despite its considerable burden and potential adverse effects on both maternal and child well-being, PPD often goes undetected and untreated.

Materials and methods

A cross-sectional study was conducted from January 2024 to March 2024 at a tertiary care center in Gorakhpur to assess PPD in 280 postpartum women. The Edinburgh Postnatal Depression Scale (EPDS) score ≥ 10 was used to confirm depression. Data collection involved a pretested, structured questionnaire. Data were analyzed using SPSS version 22 (IBM Corp., Armonk, NY). A p-value < 0.05 was considered statistically significant.

Results

The prevalence of PPD was 12.14%. Age and education were significant sociodemographic risk factors (p < 0.05). In psychosocial factors, adverse life events (p < 0.001), wishing for a male child but giving birth to a female (p = 0.01), domestic violence (p = 0.005), relationship issues, an alcoholic spouse (p = 0.01), and poor in-law relations (p < 0.001) were found to be linked to PPD. Obstetric factors such as complicated antenatal history, physical illness, cesarean section, complicated intranatal history, and postpartum complications were also found to be important factors.

Conclusion

PPD affects many women, emphasizing the need for effective measures. Initiatives like the appointment of healthcare counselors and PPD screening programs in healthcare settings are essential to detect and support affected mothers.

## Introduction

Postpartum depression (PPD) is a significant public health concern globally, affecting women across various cultures and socio-economic backgrounds. It is characterized by a spectrum of mood disturbances ranging from mild mood swings to severe depressive episodes that initiate within four weeks post childbirth and potentially persist for up to 12 months [1]. The worldwide prevalence of PPD is found to be around 17.7% [2]. As compared to developed countries, women in developing countries have shown higher rates of PPD [3]. Despite its considerable burden and potential adverse effects on both maternal and child well-being, PPD often goes undetected and untreated.

Various factors like low socioeconomic status, unemployment, lack of social or emotional support, negative attitude toward pregnancy, high parity, obstetric complications, delivery of a preterm or low birth weight baby, first-born child status, childcare stress, sleep disturbances, low self-esteem, antenatal depression or anxiety, a history of depression, disturbed marital relationships, a history of domestic abuse, significant adverse life events in the past year, neuroticism, and perfectionism are found to be strong predictors of PPD [4,5]. Clinical manifestations of PPD include sleep disturbances, changes in appetite, fear of harming the infant, excessive concerns about the baby's well-being, feeling sadness or excessive crying, self-doubt, guilt, helplessness, impaired concentration, loss of memory, loss of interest in once-beloved activities, and recurrent thoughts of death and suicide [6,7]. The impact of PPD extends beyond physical symptoms, negatively affecting the mental health and development of the babies born to affected mothers [8]. Eastern Uttar Pradesh, with its unique cultural, social, and economic dynamics, presents a distinct context for understanding and addressing postpartum mental health challenges. However, research on PPD in this region remains scarce, with limited insights into its prevalence, risk factors, and associated consequences. Therefore, this study aims to fill this critical gap by conducting a comprehensive exploration of postpartum depression within a tertiary care center located in eastern Uttar Pradesh, India.

## Materials and methods

The present cross-sectional study, spanning from January 2024 to March 2024, was conducted on postnatal females within one year of delivery at B.R.D. Medical College, Gorakhpur. Participants were selected randomly from the outpatient department (OPD) of obstetrics and gynecology during the study period. Data collection involved a pretested, structured questionnaire in conjunction with hospital records.

Inclusion criteria

The study included postnatal mothers having infants from one month to less than one year of age.

Exclusion criteria

Postpartum mothers within four weeks after delivery or not giving consent, mothers with pre-existing depression, symptoms within < two weeks postpartum to avoid confusion with postpartum blues, and postpartum women with any emergency condition were excluded from the study.

Sample size (n)

Being an observational cross-sectional study, the formula used was n = 4pq/e2, where n = sample size, p = prevalence of PPD, q = (1-p), and e = allowable error.

A recent systematic review and meta-analysis show the pooled prevalence of PPD in India at 19% [9]. The allowable error is taken to be 5%; therefore, our sample size turns out to be 244. We have taken an additional 15% to compensate for non-response and loss to follow-up. So, the final sample size was 280 participants.

Study tool

The questionnaire consisted of four sections. The first section encompassed socio-demographic information, while the second section included the questions to assess the presence of PPD. The third and fourth sections included inquiries related to psychosocial factors potentially associated with PPD. The Edinburgh Postnatal Depression Scale (EPDS) was used (Hindi version) to assess PPD. EPDS is an easy and effective tool to screen PPD cases [10]. EPDS featured 10 questions focusing on common depression symptoms. Each question employed a four-point scale, ranging from 0 to 3, resulting in a minimum score of 0 and a maximum score of 3 for each question. A cumulative score of 10 or more confirmed the presence of depression, whereas a score of 9 or less indicated the absence of PPD, categorizing the female participants as normal.

Ethical clearance

Ethical clearance was obtained from the Institutional Human Ethical Committee, Baba Raghav Das Medical College, Gorakhpur (Approval No.: 37/IHEC/2024) before the start of the study.

Statistical analysis

Data were entered in Excel (Microsoft Corporation, Redmond, WA), and after data cleaning, they were coded, imported, and analyzed using IBM SPSS Statistics version 22.0 (IBM Corp., Armonk, NY). Descriptive analysis was undertaken to present an overview of the findings as proportions and percentages in tabular form. The chi-square test, or Fisher exact test, was performed for categorical variables to determine the associations between PPD and other relevant factors. A p-value < 0.05 was considered statistically significant.

## Results

In the current study, a total of 280 eligible participants were recruited and completed the study. These were postpartum women within one year of attending the outpatient department of obstetrics and gynecology. The prevalence of PPD in our study was 12.14% (Table 1).

**Table 1:** Prevalence of postpartum depression among study participants (n = 280)

Postpartum depression	Frequency	Percent (%)
Present	34	12.14
Absent	246	87.86

The mean age of the study population was 24.1 ± 4.1 years, and 58.8% of women with PPD fell into the age group of <25 years (p < 0.001). Furthermore, 58.8% of women with postpartum depression had no formal education, which is also statistically significant (p = 0.02). The majority of PPD cases were housewives (94.1%). A significant proportion of PPD cases occurred in women from rural backgrounds (82.4%), whereas only 17.6% were from urban settings. Most women with PPD were financially dependent (94.1%), with only one financially independent woman found to be depressed. Among women with PPD, 74.3% identified as Hindu by religion, and only 29.4% belonged to other religions, thus not being established as a predictor of PPD (Table 2).

**Table 2:** Postpartum depression based on the demographic profile of the study participants (n = 280) P-value: significant value.

Parameters		Total participants, n (%)	Depressed, n (%)	Not depressed, n (%)	p-value
Age (years)	>25	202 (72.2)	14 (6.9)	188 (93.1)	<0.001
	<25	78 (27.8)	20 (25.6)	58 (74.4)	
Education	Uneducated	96 (34.3)	20 (20.8)	76 (79.2)	0.02
	Educated	184 (65.7)	14 (7.6)	170 (92.4)	
Occupation	Housewife	270 (96.4)	32 (11.8)	238 (88.2)	0.58
	Working lady	10 (3.6)	2 (20)	8 (80)	
Residence	Rural	224 (80)	28 (12.5)	196 (87.5)	0.7
	Urban	56 (20)	6 (10.7)	50 (89.3)	
Religion	Hindu	208 (74.2)	24 (11.5)	184 (88.5)	0.71
	Others	72 (25.8)	10 (13.9)	62 (86.1)	

Table 3 shows the PPD based on psychosocial factors. The majority (70.6%) of women with PPD had a history of adverse life events, which was statistically highly significant (p < 0.001). Additionally, 58.8% of these women desired a male child but delivered a female child, which was also a statistically significant predictor (p = 0.01). Similarly, 52.9% of women with PPD had a history of domestic violence, which was statistically significant (p = 0.005). Notably, 70.6% of women with PPD reported an inadequate relationship with their in-laws, a statistically significant predictor (p = 0.001). Furthermore, 52.9% of women with PPD had husbands with a history of alcohol or psychotropic drug use, and this was also a statistically significant predictor (p = 0.01).

**Table 3:** Postpartum depression based on psychosocial factors (n = 280) P-value: significant value.

Psychosocial factors		Total participants	Depressed, n (%)	Not depressed, n (%)	p-value
Psychosocial factors-I					
Adverse life events	Yes	54 (19.3)	24 (44.4)	30 (55.6)	0.001
	No	226 (80.7)	10 (4.4)	216 (95.6)	
Pre-existing psychiatric illness	Yes	22 (7.9)	0 (0)	22 (100)	0.4
	No	258 (92.1)	34 (13.18)	224 (86.82)	
Family history of psychiatric illness	Yes	28 (10)	6 (21.4)	22 (78.6)	0.11
	No	252 (90)	28 (11.1)	224 (91.1)	
Family history of postpartum depression	Yes	08 (2.9)	0 (0%)	8 (100)	0.06
	No	272 (97.1)	34 (12.5)	238 (87.5)	
Psychosocial factors-II					
Total no. of girl child	1	228 (81.4)	26 (11.4)	202 (88.6)	0.5
	≥1	52 (18.6)	8 (15.4)	44 (84.6)	
Total no. of male child	0	206 (73.6)	30 (14.5)	176 (85.5)	0.314
	At least one	74 (26.4)	6 (8.1)	68 (91.9)	
Pressure to have a male child	Yes	142 (50.7)	20 (14.1)	122 (85.9)	0.48
	No	138 (49.3)	14 (10.1)	124 (89.9)	
Wanted a male but gave birth to a female	Yes	90 (32.1)	20 (18.9)	70 (81.1)	0.011
	No	190 (67.9)	14 (8)	176 (92)	
Psychosocial factors-III					
Domestic violence	Yes	78 (27.9)	18 (23.1)	60 (76.9)	0.005
	No	202 (72.1)	16 (8)	186 (92)	
Closely attached to the spouse	Yes	216 (77.1)	16 (7.4)	200 (92.6)	0.001
	No	64 (22.9)	18 (28.1)	46 (71.9)	
Alcohol or psychotropic drug intake by spouse	Yes	78 (27.9)	18 (23.1)	60 (76.9)	0.01
	No	202 (72.1)	16 (7.9)	186 (92.1)	
Adequate relationship with parents	Yes	246 (87.9)	30 (88.2)	216 (87.8)	0.1
	No	34 (12.1)	4 (11.8)	30 (12.2)	
Adequate relationship with in-laws	Yes	182 (65)	10 (5.5)	172 (94.5)	<0.001
	No	98 (35)	24 (24.5)	74 (75.5)	

The relationship between obstetric factors and PPD is given in Table 4. The majority (70.6%) of women with PPD had complicated antenatal histories, which proved to be a statistically significant predictor (p < 0.001). A significant 84.4% of women with PPD had a history of other physical illnesses, establishing this as a statistically significant predictor (p < 0.001). Additionally, 58.8% of women with PPD underwent a cesarean section as their mode of delivery, a statistically significant predictor (p < 0.001).

**Table 4:** Relationship of obstetric factors with PPD (n = 280) NVD: normal vaginal delivery; PPD: postpartum depression; p-value: significant value; P, parity.

Obstetric factors		Total participants, n (%)	Depressed, n (%)	Not depressed, n (%)	p-value
Parity	P_1_	154 (55)	14 (9.1)	140 (90.9)	0.221
	P_≥2_	126 (45)	20 (15.9)	106 (84.1)	
Abortion	Present	68 (24.3)	10 (14.7)	58 (85.3)	0.6
	Absent	212 (75.7)	24 (11.3)	188 (82.7)	
Live issues(number)	1	166 (59.3)	22 (13.2)	144 (86.8)	0.63
	≥1	114 (40.7)	12 (10.5)	102 (89.5)	
Prenatal history	Uncomplicated	224 (80)	10 (4.5)	214 (95.5)	<0.001
	Complicated	56 (20)	24 (42.9)	32 (57.1)	
History of other medical illnesses	Present	58 (20.7)	28 (48.3)	30 (51.7)	<0.001
	Absent	222 (79.3)	6 (2.7)	216 (97.3)	
Mode of delivery	NVD	200 (71.4)	14 (7)	186 (93)	<0.001
	Cesarean	80 (28.6)	20 (25)	60 (75)	
Intranatal history	Uncomplicated	240 (85.7)	16 (6.7)	224 (93.3)	0.001
	Complicated	40 (14.3)	18 (45)	22 (55)	

## Discussion

Our study explores PPD among women within one year postpartum in a tertiary care center in eastern Uttar Pradesh, India. Several noteworthy findings have emerged. Firstly, the prevalence of PPD in our study was 12.14%, indicating a significant proportion of women experiencing postnatal depressive symptoms within the region, which aligns closely with a study by Chandran et al., who reported an incidence of 11% [11]. Various reports have indicated a mean prevalence rate of PPD ranging from 10% to 15% in different countries [12-14].

Relationship of sociodemographic factors with PPD

In our study, a higher proportion of PPD cases (58.8%; 20/34) were observed in individuals aged less than 25 years (p < 0.001), possibly influenced by early marriage practices in the region where younger women might not be adequately prepared to manage the responsibilities of both motherhood and household duties. This trend is consistent with findings from other studies, all of which underscore the significance of this age factor in PPD development [15-17].

In terms of education, our study demonstrated that 58.8% (20/34) of women with no formal education experienced depression. This association was found to be statistically significant (p = 0.02). This aligns with some previous studies, as illiterate women may face challenges in achieving economic and emotional independence [11,18,19]. The majority of women in our study were housewives, consistent with findings in other Indian studies [11,18], whereas Western studies often report a significant number of employed women [20]. This may reflect a low self-esteem among women who were not employed or a significant role change for women previously employed, but who, after childbirth, do not have future employment.

Most of the participants in our study came from rural backgrounds. However, this cannot be conclusively established as a predictor of depression, as 80% of the total study population came from rural backgrounds, and to assess the effect of residence on PPD, a comparable number of participants from urban areas would be necessary. Some studies have reported that women in rural areas are at an increased risk of developing PPD, and various factors, including differences in socioeconomic status, fertility, and family organization between rural and urban areas, may be responsible for that [18,21]. Concerning financial dependence, the vast majority of participants in our study (96.4%) were financially dependent, preventing an assessment of its relation to PPD. Financial independence typically affords women the liberty to care for themselves and their newborns, reducing their susceptibility to depression [15]. However, religion did not emerge as a predictor of PPD in our analysis, indicating that other factors may have a more substantial influence on postpartum mental health outcomes within this population.

Relationship of psychosocial factors I with PPD

In our study, among the 34 depressed women, 24 reported a history of adverse life events, including financial constraints, the unavailability of their husbands during the antenatal and postpartum periods, the death of a baby, and the death of a husband. It is noteworthy that the death of loved ones, ailing parents, husbands being posted to field areas, and financial constraints are common stressors experienced by depressed mothers. Pregnancy and childbirth, in themselves, can be inherently stressful life events, potentially contributing to depression [6,12,18]. In our study, none of the depressed women had pre-existing psychiatric illnesses. However, contrasting results from a study suggest that women with pre-existing psychiatric conditions are more susceptible to developing PPD, possibly because the additional stress of childbirth can be particularly challenging for these women [22].

No significant association (p = 0.11) was found between a family history of psychiatric illness and PPD. This differs from the findings in other studies, which consistently reported a family history of depression as a risk factor for PPD [19,21,23]. The variation in results between our study and others may stem from the underreporting of family history of psychiatric illness, often due to a lack of knowledge or social reasons discouraging disclosure. Regarding a family history of postpartum depression, the present study did not identify any significant association (p = 0.06) between PPD and a family history of PPD. Only one study reported that women with a family history of PPD are at a higher risk of developing the condition [15].

Child gender relationship with postpartum depression (psychosocial factors II)

The present study did not indicate any association (p > 0.05) between PPD and the number of girl children, possibly because most participants in the study group had only one girl child, with only four participants having two or more girl children. Conversely, some studies reveal that the presence of more than one girl child in the family is still considered a disadvantage in various cultures and socioeconomic groups [12,24].

In the present study, pressure to have a male child did not show any association (p = 0.31) with the development of PPD. Most of the women had only one child, and only four women had two or more girl children. However, some Indian studies revealed that pressure to have a male child was significantly relevant to the development of PPD [16,18]. Pressure from in-laws on women to deliver a male child is tremendous in many parts of the country, and not fulfilling this family wish can lead to an environment that increases the risk of PPD.

In the current study, 58.8% (20/34) of depressed women desired a male child but delivered a female, and this was identified as a significant risk factor for postpartum depression (p-value = 0.01). In South Asian culture, the birth of a girl child when a male child is desired is often considered a misfortune. Some Indian studies also found this risk factor to be significant, although a study by Gupta et al. did not find it significant in the development of PPD [12,15,17].

Family relationships in postpartum depression (psychosocial factors III)

In our study, domestic violence was found to be a significant risk factor (p = 0.005) for the development of PPD. Domestic violence, by imposing additional stress, contributes to the increased incidence of PPD in these women. Other Indian studies also found domestic violence to be a significant risk factor for developing PPD [17,25]. In our study, it was found that women who were not closely attached to their spouses had a higher likelihood of developing PPD (p = 0.001). Close attachment to the spouse can cultivate a feeling of contentment, potentially explaining the finding that improper attachment to one's partner is a risk factor for the development of postpartum depression. This aligns with the results of other Indian studies [12,15].

Regarding the intake of alcohol and psychotropic drugs by husbands, our study revealed that 52.9% of women who developed PPD had a positive history of alcohol and psychotropic drug abuse by their husbands (p = 0.01). Substance abuse by the husband causes additional stress for women during pregnancy and the postpartum period due to a lack of emotional and financial support. This finding is consistent with other studies [15,17].

In the context of the relationship with parents, the present study did not find inadequate parental relationships to be a significant risk factor for the development of PPD (p = 0.10), as most of the postpartum females had adequate parental support during and after delivery. However, this was found to be significantly relevant to the development of PPD in other Indian studies [12,15]. Furthermore, the study indicates that 70.6% of women in the depressed group did not have an adequate relationship with their in-laws, showing that inadequate relationships with in-laws significantly increased the risk of PPD (p = 0.001), a pattern also observed in some other studies [12,15].

Obstetric factors in postpartum depression

In this study, parity, abortion, and the number of live issues did not find any significant association (p = 0.05) with the development of PPD. This might be because a large number of women in the study group were primiparous, with only one live issue and no history of abortion. Similar results were observed in other studies [26,27]. In contrast, complications during pregnancy were found to be a significant risk factor for the development of PPD, with 70.6% of depressed women having a complicated antenatal history (p < 0.001). Furthermore, the study revealed that women with a history of other medical illnesses, such as anemia, generalized tonic-clonic seizures, heart disease, asthma, and sexually transmitted infections, were more likely to develop postpartum depression (p < 0.001). This could be because they already had the mental stress of dealing with a particular disease, and when combined with the stress of pregnancy, it increased the risk of postpartum depression. Providing respectful maternity care may further help in reducing the chances of PPD. [28].

The findings of the present study indicated that women who experienced complications during labor were more likely to develop PPD (p < 0.001). In the context of the numerous hormonal and bodily changes associated with childbirth, complications during delivery can add to the stress of the process, potentially explaining the increased risk of PPD. This aligns with the results of other studies [29,30]. Regarding the mode of delivery, in the present study, 20 out of 34 depressed women had a history of emergency cesarean section as the mode of delivery due to various maternal and fetal complications. As a result, emergency cesarean sections were reported as a significant contributor to PPD (p < 0.001). This might be attributed to the severe mental stress associated with both the complications themselves and the unexpected nature of emergency surgery. However, another study did not show a relationship between the mode of delivery and PPD [24].

Recently, concern has been expressed about the high prevalence of depression in developing countries and the need to develop cost-effective intervention strategies. Women who have certain risk factors have a significantly increased risk of experiencing the illness. The study thus signifies the importance of identifying postpartum depression, more so because none of these women had sought help for these symptoms, although they were functionally impaired. A higher prevalence of PPD among younger age women found in the study signifies interventions to be focused on providing comprehensive education and support for these high-risk women. Further interventions should focus on vocational training and providing employment opportunities, empowering women to attain financial independence. Ensuring access to quality antenatal and postnatal care, regular screening for mental health issues, providing psychosocial support for women during pregnancy and childbirth, and culturally sensitive care practices are also crucial in promoting positive maternal mental health outcomes. Engaging community leaders, healthcare providers, and local help groups in raising awareness and providing support to at-risk women can enhance early detection and effective management of PPD.

Limitations

The study has a few limitations that warrant consideration. Firstly, its cross-sectional design restricts the ability to establish causal relationships between identified risk factors and PPD, necessitating longitudinal investigations for more robust conclusions. Additionally, the sampling bias inherent in recruiting participants solely from a single tertiary care center might limit the generalizability of findings to the broader population of postpartum women. Furthermore, the exclusion criteria, such as excluding women with pre-existing depression, might underestimate the true prevalence of PPD.

## Conclusions

PPD has a considerable burden on postpartum women. Sociodemographic factors such as younger age, lack of formal education, and rural background have emerged as significant predictors of PPD. Psychosocial factors, including adverse life events, domestic violence, and inadequate spousal relationships, were strongly associated with PPD, highlighting the need for comprehensive support systems for postpartum women. Obstetric factors such as antenatal complications and mode of delivery also contributed to PPD risk. Further research is warranted to explore treatment-seeking behavior and intervention effectiveness to inform targeted strategies for PPD prevention and management.
